# Supplementary figures and images for: Intestinal permeability and peripheral immune cell composition are altered by pregnancy and adiposity at mid- and late-gestation in the mouse
Source: PLoS One. 2023 Aug 7;18(8):e0284972. doi: 10.1371/journal.pone.0284972 (PMC10406227; doi:10.1371/journal.pone.0284972)

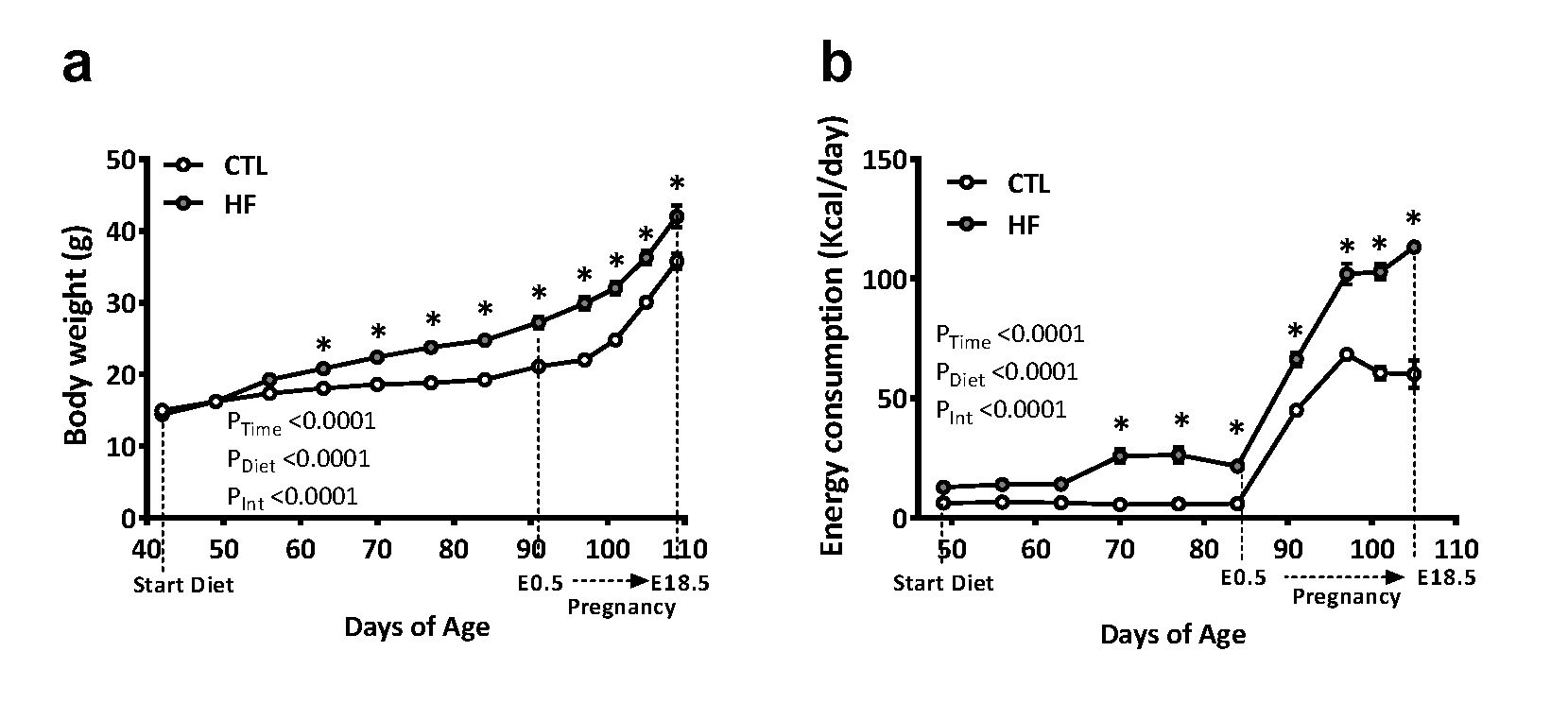

Supplement: S1 Fig — Female mice were fed a standard chow control (CTL; n = 7–12) diet or high fat (HF; n = 9–13) diet for 6 weeks, mated with CTL-fed mice, and maintained on their diet throughout pregnancy. (a) maternal body weight was measured at the start of diet allocation (W0), weekly (W1-W6) and during pregnancy (at gestational days E0.5, E6.5, E10.5, E14.5, E18.5); (b) maternal energy consumption (kilocalories per day). Data in line graphs are shown as mean ± SEM. *P<0.05. (TIF) [file pone.0284972.s001.tif]

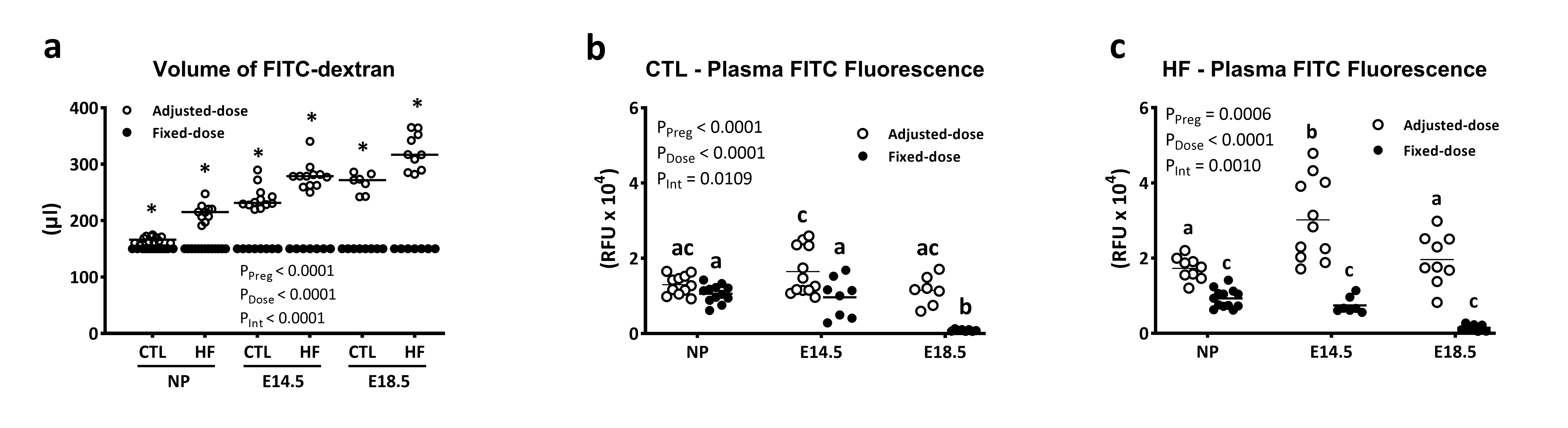

Supplement: S2 Fig — Validation experiments were performed to assess the appropriate dose of FITC-dextran by oral gavage to evaluate in vivo intestinal permeability in non-pregnant female mice, and pregnant E14.5 and E18.5 dams, fed a standard chow diet (CTL; n = 7–12) or high fat diet (HF; n = 9–14). a) volume of FITC -dextran administered in fixed-dose compared to weight adjusted-dose; b) plasma FITC fluorescence in CTL diet-fed non-pregnant female mice and pregnant dams at 4 hours post gavage; c) plasma FITC fluorescence in HF-fed non-pregnant female mice and pregnant dams at 4 hours post gavage. Data are shown as scatter dot plots and the center line indicates the median. Each data point is an individual mouse. Box plots with different letters indicate statistical significance of P<0.05. (TIF) [file pone.0284972.s002.tif]

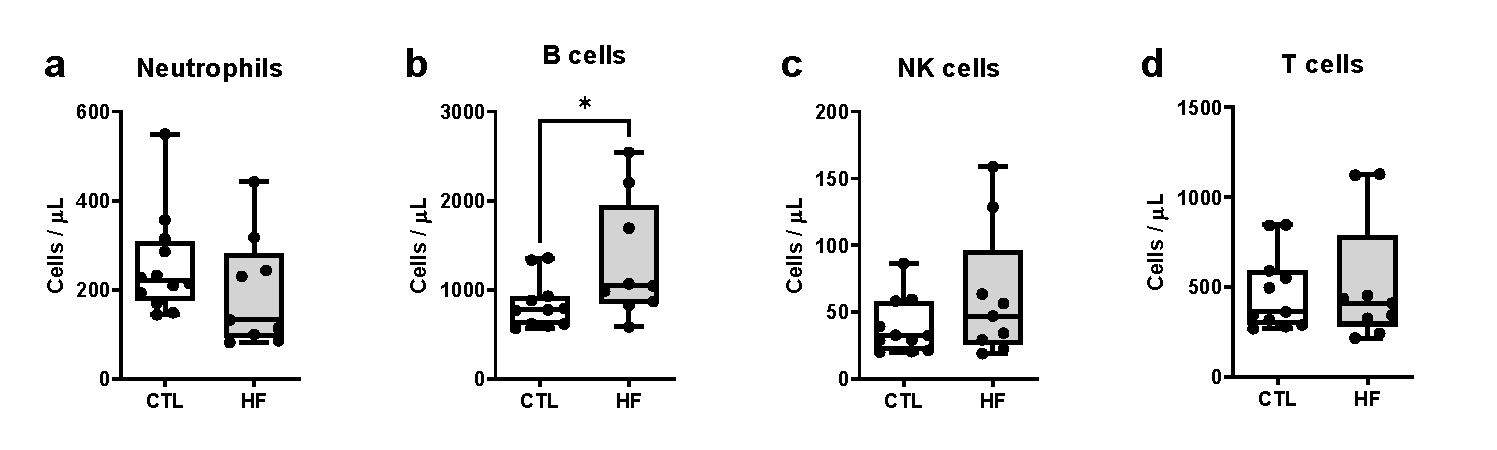

Supplement: S3 Fig — Maternal peripheral blood and immune cell populations of standard chow control-fed (CTL; n = 11–12) dams and high fat-fed (HF; n = 9) dams were assessed by flow cytometry at E14.5. Absolute cell counts of: (a) neutrophils, (b) B cells, (c) NK cells, and (d) T cells. Each data point indicates an individual mouse. Data are presented as box and whisker plots, min to max, where the centre line shows the median. *P<0.05. (TIFF) [file pone.0284972.s003.tiff]
