# Supplementary material for: Intestinal permeability and peripheral immune cell composition are altered by pregnancy and adiposity at mid- and late-gestation in the mouse
Source: PLoS One. 2023 Aug 7;18(8):e0284972. doi: 10.1371/journal.pone.0284972 (PMC10406227; doi:10.1371/journal.pone.0284972)
Supplement: Attachment — Submitted filename: Revewer_Responses_PONE-D-22-25312_20230321.pdf [file pone.0284972.s004.pdf]

Deborah M Sloboda, Professor

Department of Biochemistry and Biomedical Sciences
1200 Main Street West;
Hamilton, Ontario, Canada, L8N 3Z5
Phone 905.525.9140
Fax 905.522.9033
http://www.fhs.mcmaster.ca/biochem

March 16, 2023

Re: *PONE-D-22-25312 "Intestinal permeability and peripheral immune cell composition are altered by pregnancy and adiposity at mid- and late-gestation in the mouse"*

Dear Dr. Torrens and the Editorial Board and Reviewers for *PLOS ONE*,

Thank you for the supportive and constructive review of our manuscript. We have addressed the Academic Editor and Reviewers' comments below in a point-by-point manner and have made corresponding revisions to the manuscript where appropriate.

The manuscript has also been reviewed and revised as requested to ensure its contents, style and formatting comply with the latest PLOS ONE journal policies. We have also reviewed and revised the methods section, funding statement, data availability, and reference list as requested. Further details are below.

Journal Requirements:

When submitting your revision, we need you to address these additional requirements.

1. Please ensure that your manuscript meets PLOS ONE's style requirements, including those for file naming. The PLOS ONE style templates can be found at
https://journals.plos.org/plosone/s/file?id=wjVg/PLOSOne_formatting_sample_main_body.pdf
and
https://journals.plos.org/plosone/s/file?id=ba62/PLOSOne_formatting_sample_title_authors_affiliations.pdf

We believe we have made appropriate changes to the author, main body formatting, and file naming as outlined by the PLOS ONE style templates.

2. To comply with PLOS ONE submissions requirements, in your Methods section, please provide additional information regarding the experiments involving animals and ensure you have included details on (1) methods of sacrifice, (2) methods of anesthesia and/or analgesia, and (3) efforts to alleviate suffering.

Additional information has been added to the methods to comply with the above requirements (lines 119-120).

3. Thank you for stating in your Funding Statement:

“This work was funded by a team grant from the Canadian Institutes of Health Research (CIHR) led by DMS. TAR was supported by a McMaster Institute for Research on Aging Postdoctoral Fellowship and a Michael G. DeGroote Fellowship Award. JAB was supported by a Queen Elizabeth II Scholarship in Science and Technology. Work in the Bowdish laboratory is supported by the McMaster Immunology Research Centre and the M. G. DeGroote Institute for Infectious Disease Research. DMEB and DMS are/were supported by the CIHR and are Canada Research Chairs.”

Please provide an amended statement that declares *all* the funding or sources of support (whether external or internal to your organization) received during this study, as detailed online in our guide for authors at http://journals.plos.org/plosone/s/submit-now. Please also include the statement “There was no additional external funding received for this study.” in your updated Funding Statement. Please include your amended Funding Statement within your cover letter. We will change the online submission form on your behalf.

We have reviewed the Funding Statement and have amended the text below to include the requested information:

This work was funded by a team grant from the Canadian Institutes of Health Research (CIHR) led by DMS. TAR was supported by a McMaster Institute for Research on Aging Postdoctoral Fellowship and a Michael G. DeGroote Fellowship Award. JAB was supported by a Queen Elizabeth II Scholarship in Science and Technology. KMK and EY were supported by a Farncombe Digestive Health Research Institute Student Fellowship. BKEK and CJB were supported by a CIHR Canada Graduate Scholarships – Master’s (CGS-M) award. PAJ was supported by a Thomas Neilson Scholarship, Fred & Helen Knight Enrichment Award, and an Ontario Graduate Scholarship. FFA was supported by a CIHR Fellowship. JDS holds a Canada Research Chair in Metabolic Inflammation. DMEB holds a Canada Research Chair in Aging and Immunity. DMS was funded by a Canada Research Chair in Perinatal Programming. There was no additional external funding received for this study.

4. We note that you have stated that you will provide repository information for your data at acceptance. Should your manuscript be accepted for publication, we will hold it until you provide the relevant accession numbers or DOIs necessary to access your data. If you wish to make changes to your Data Availability statement, please describe these changes in your cover letter and we will update your Data Availability statement to reflect the information you provide.

We have chosen to upload our data to FigShare.

Data are accessible at 10.6084/m9.figshare.22285792.

5. Please review your reference list to ensure that it is complete and correct. If you have cited papers that have been retracted, please include the rationale for doing so in the manuscript text, or remove these references and replace them with relevant current references. Any changes to the reference list should be mentioned in the rebuttal letter that accompanies your revised manuscript. If you need to cite a retracted article, indicate the article’s retracted status in the References list and also include a citation and full reference for the retraction notice.

The reference list has been reviewed to ensure it is complete and correct. No papers have been cited that have been retracted. We noticed that there were 3 duplicate references (Aghaeepour et al, 2017; Breznik et al., 2021; Ingvorsen et al., 2014), and those duplicates have been removed. Some additional references have been added in response to reviewer comments, and are noted with the comments below.

Reviewer #1:

This is an interesting study by Ribeiro and colleagues, who have examined the structural morphology of mouse intestine under normal diet and high fat conditions at different stages in pregnancy This is a well written and well designed study with interesting observations on the dynamic changes in intestinal structure and permeability under the experimental conditions.

Abstract.
Provide statement on the inferences from the findings – why are they important (currently, there is only a summary of the data).

We thank the reviewer for the suggestion and have inserted text within the abstract stating:

> “Moreover, it remains unknown how pregnancy and diet can interact to alter intestinal barrier function.” (lines 32-33)

and

> “Maternal adaptations in pregnancy are thus vulnerable to excess maternal adiposity, which may affect maternal and child health.” (lines 48-49)

Introduction
Place rework the sentence ‘This barrier is necessary to prevent systemic dissemination of luminal contents including the intestinal microbiota.’ It suggests total breakdown of the gut barrier and opposed to more subtle modulation of permeability.

This sentence has been rewritten (see lines 67-69):

> “Intestinal microbial dysbiosis and accompanying reductions in barrier function have been associated with gradual dysregulation of whole-body metabolism.”

A new reference – Lam *et al.* 2012 has been added (https://doi.org/10.1371/journal.pone.0034233).

The sentence ‘Thus, maintenance of the intestinal barrier is essential to prevent both local and systemic effects of HF diet intake.’ suggests a ‘cause and effect’ relationship between gut permeability and body-wise effects of a diet high in fat. While it may be part of the story, there are other factors involved too, which should be acknowledged.

We thank the reviewer for the suggestion. We have revised the sentence so it is less ‘cause and effect’ focussed but still provides a summary of the preceding information on intestinal barrier function and local and systemic changes that are observed in diet-induced obesity (See lines 85-86):

> "Thus, loss of intestinal barrier function is likely an important contributor to local and systemic effects of HF diet intake.”

Reviewer #2:

This study verified that pregnancy induced changes in the physical structure and function of maternal intestinal epithelium, which had a systemic effect on immunity, and maternal obesity caused by the HF diet further aggravated this effect. Therefore, I think this study is of great significance and suggest that it be revised and published.

1、It is not necessary to introduce statistical methods below each figure, except for special methods

The figure caption text has been revised as requested to remove statistical methods as they are described fully within the methods. (See lines 25-255; 274-277; 299-301; 324-326; 335-337; 376-381; 679-681; 691-694; 701-703).

2、Inflammatory factors in serum have a close relationship with intestinal permeability. It is suggested that some inflammatory factors be supplemented in serum.

We have interpreted ‘supplemented’ as ‘measured’. We agree with the reviewer that intestinal permeability has been associated with systemic inflammation, which may be reflected in the composition of serum inflammatory factors like cytokines, and/or cellular components of immunity like monocytes.

Using the same high fat diet model in pregnancy, we have previously measured (ref. 23) serum cytokines at E14.5 in chow and high-fat fed dams (see below from Figure 4 of Wallace *et al.*, 2019: 10.1038/s41598-019-54098-x). We detected no statistically significant differences in levels of these serum inflammatory factors between chow and HF-fed dams.

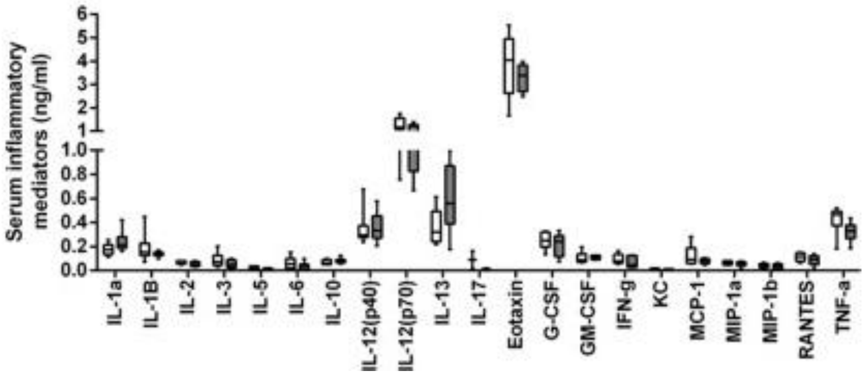

However, we previously observed that levels of bacterial products LPS (lipopolysaccharide) and MDP (muramyl dipeptide) were elevated in serum of HF-fed dams compared to chow-fed dams at E18.5 (using the same diet model) (see right and Figure 4 of Gohir & Kennedy *et al.*, 2019: https://doi.org/10.1113/JP277353).

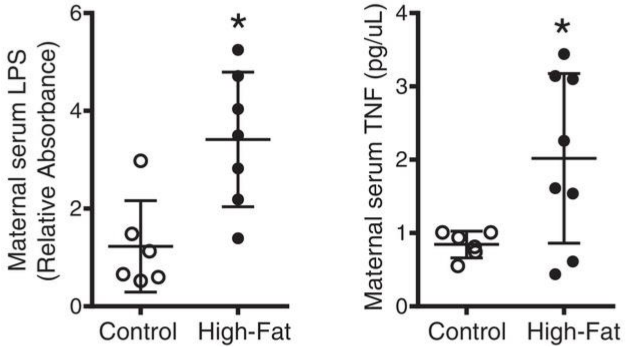

We realize that these data were not explicitly cited in the current manuscript, so text has been added as follows in the introduction as part of the justification for this study: Lines 95-96:

> "..., and we have also observed elevated serum levels of bacterial call wall components lipopolysaccharide (LPS) and muramyl dipeptide (MDP) in HF-fed dams late in gestation (14)."

The reference for Gohir & Kennedy *et al.*, 2019 (https://doi.org/10.1113/JP277353) was included.

We have also acknowledged that we did not measure serum cytokines or bacterial products in this particular manuscript within a new section on study limitations within the discussion, as requested by Reviewer 3 (lines 443-447), and referenced both of the studies above.

> "…We have previously assessed serum inflammatory mediators including cytokines at E14.5 and bacterial LPS and MDP at E18.5 using the same mouse model (14, 23), but we did not repeat those measures in context of our assessments of intestinal permeability and peripheral cellular immunity within this study…".

3、Some figures are marked as ' * ', some are marked as ' abc ', and some are not marked. It is suggested to use a unified symbol to mark the significance, and add that there is no significant difference if there is no mark.

In the figures in this manuscript, statistical significance between groups is indicated by asterisks when two-group analyses *i.e.,* Student's t test or Mann-Whitney U test, were used. In contrast, two-way ANOVA analyses are reported with main effects and post-hoc similarities and differences are indicated by letters. If two groups share the same letters, then they are similar, whereas if they have different letters, they are significantly different (*i.e.,* P is at least <0.05). We regrettably cannot assign P values based on letter combinations, as statistically significant differences may not be the same between dissimilar letter pairs.

We believe the current formatting is not inconsistent with journal requirements but would be happy to change the formatting if the Editor deems it appropriate.

4、It is recommended that ' ab ' represents P < 0.05 and ' AB ' represents P < 0.01.

Please see the above response for comment 3.

5、The abstract is a high-level summary of the full text.Although there is no clear definition, the general abstract must contain key words, and 'maternal adaptation' does not appear in the abstract.

We thank the reviewer for this observation. We have included "maternal adaptation" in the abstract (line 31): "Despite significant physiological maternal adaptations that occur across gestation…"

Reviewer #3:
This well-written manuscript describes novel data that addresses a gap in the current literature at the intersection of gut structure and function, high fat diets, and pregnancy. T

Questions and concerns that might strengthen the manuscript.
• In the introduction (line 82), the author references unpublished data which does not appear to be appropriately cited.

This data referenced was included within a manuscript that was in review at the time of submission of this manuscript. That manuscript has since been accepted for publication. The appropriate reference (see below) has been added (line 82 – ref. 32).

Breznik JA, Jury J, Verdu EF, Sloboda DM, Bowdish DME. Diet-induced obesity alters intestinal monocyte-derived and tissue-resident macrophages and increases intestinal permeability in female mice independent of TNF. Am J Physiol Gastrointest Liver Physiol. 2023 Feb 7. doi: 10.1152/ajpgi.00231.2022. Epub ahead of print. PMID: 36749921.

• In the methods (line 102), there is no justification for the use of a 60% HF diet as opposed to using a 45% HF diet, which in the literature appears to be more physiologic for translation to humans. Exclusion of an intermediate HF diet, 45% HF diet, also limits the opportunity for exploring dose-dependent responses.

We thank the reviewer for this observation, and we agree that adding context for choosing a 60% HF diet is important. We chose that diet because this study builds on our previous work that has used that diet to obtain a phenotype of excess adiposity/obesity with accompanying metabolic dysregulation. We recognize that there has been discussion in the literature that a 45% kcal from fat diet may be more physiologically translatable to humans, and agree with the reviewer that including an 'intermediate' high fat diet would offer opportunities for exploring dose-dependent responses. Currently we are conducting studies using both the 45% and 60% diets.

We would also like to point out that the commonly used 45% / 60% diets (from ResearchDiets) have differences in relative dietary fat composition (i.e., there is more soybean oil of total fat in the 60% diet) and sucrose content. Even studies that attempt to identify the 'ideal' HF diet recognize that effects of HF diet with specific mouse models may differ. Mouse strain, sex, age, and health status affect the response of mice to obesogenic diets, and the composition of the fat and carbohydrate content, and macronutrient and micronutrient components within the diet can also affect the development of obesity and other effects of high fat diet intake (see https://doi.org/10.1186/s13098-021-00647-2 and https://doi.org/10.1038/oby.2007.608). Thus, understanding specific effects of HF diet in a specific lab setting and mouse model type, while acknowledging limitations, is essential to develop ways to manipulate that model.

We have added the following text into the Methods, which we hope addresses the Reviewer's concerns (lines 110-112):

"We chose a standard high fat diet, of 60% kcal fat, based on our previous work using this model in both non-pregnant female mice (32, 33) and in pregnancy (14, 15, 40)."

References:
14. Gohir W, Kennedy KM, Wallace JG, Saoi M, Bellissimo CJ, Britz-McKibbin P, et al. High-fat diet intake modulates maternal intestinal adaptations to pregnancy and results in placental hypoxia, as well as altered fetal gut barrier proteins and immune markers. The Journal of physiology. 2019;597(12):3029-51.
15. Wallace JG, Bellissimo CJ, Yeo E, Fei Xia Y, Petrik JJ, Surette MG, et al. Obesity during pregnancy results in maternal intestinal inflammation, placental hypoxia, and alters fetal glucose metabolism at mid-gestation. Scientific reports. 2019;9(1):17621.
32. Breznik JA, Jury J, Verdu EF, Sloboda DM, Bowdish DME. Diet-induced obesity alters intestinal monocyte-derived and tissue-resident macrophages and increases intestinal permeability in female mice independent of TNF. Am J Physiol Gastrointest Liver Physiol. 2023 Feb 7. doi: 10.1152/ajpgi.00231.2022. Epub ahead of print. PMID: 36749921.
33. Breznik JA, Foley KP, Maddiboina D, Schertzer JD, Sloboda DM, Bowdish DME. Effects of Obesity-Associated Chronic Inflammation on Peripheral Blood Immunophenotype Are Not Mediated by TNF in Female C57BL/6J Mice. ImmunoHorizons. 2021;5(6):370-83.
40. Gohir W, Whelan FJ, Surette MG, Moore C, Schertzer JD, Sloboda DM. Pregnancy-related changes in the maternal gut microbiota are dependent upon the mother's periconceptional diet. Gut microbes. 2015;6(5):310-20.

• In the methods (line 114), there is no justification for why the gestational timepoints of E14.5 and E18.5 were chosen.

We thank the reviewer for this observation. Within the abstract (lines 33-34) we identified that in C57BL/6 mouse models of pregnancy, embryonic day (E)14.5 is mid-gestation, while E18.5 is late/term-gestation. Pregnancy is typically <20 days in C57BL/6 mice. We recognize that this was not stated within the Methods, and have amended text (highlighted) within the methods (line 125):

"Subsets of pregnant mice at E14.5 (mid-gestation) or E18.5 (late-gestation) were allocated for…"

• In the methods (line 120), the use of the FITC assay appears to cloud the picture given high doses of the biomarkers were needed to illicit a response in the pregnant cohort. This in part may be due to known dysmotility caused by relaxin during pregnancy leading to slower transit in the gut. High fat diets have also been shown to slow down motility in the gut and may play a role in the varied results of this assay. As a future approach, the authors might consider ex vivo Ussing chamber permeability studies, which will not be clouded by differing body weights or altered motility.

We believe we have addressed the Reviewer's concerns about FITC-dextran assays through our validation of a new approach for the assay that considers dysmotility and differences in body weight by using a time course and dose adjusted by body weight. As stated within the manuscript (lines 208-209):

"…recent work has highlighted the importance of considering differences in gut transit time by mouse strain and sex (46, 47, 50-53). As maternal body weight

changes dramatically over the course of gestation, and adiposity/ obesity has been reported to alter gut transit (54), and gut transit time is increased late in pregnancy (55), we set out to determine the optimal approach for assessing whole-gut permeability in CTL and HF-fed NP and pregnant female mice."

Our validation experiments showed that a fixed dose, as suggested by the reviewer, was inappropriate for the above reasons, and led us to use an adjusted dose of FITC-dextran by body weight. We fasted mice for 6 hours prior to the assay and our experiments incorporated sampling over 4 hours to assess if peaks in fluorescence were different over time (changes in time to peak fluorescence between animal groups may be associated with differences in gut motility). Therefore, we did consider both differing body weights and altered motility in our experimental approach for the *in vivo* permeability assay. The final dose of FITC-dextran that we used was 0.5 mg/g body weight (lines 215-217), which is lower than that of other publications (e.g. 0.6 mg/g body weight used by Woting & Blaut, 2018; 10.3390/nu10060685).

However, we agree with the reviewer that *ex vivo* Ussing chamber experiments are the 'gold standard' to measure tissue-localized intestinal permeability. We have previously published data on *in vivo* permeability using a fixed dose of 150 µL or 200 µL of 80mg/mL FITC-dextran, with corroboration of those data using Ussing chambers. Specifically, we found using a fixed FITC-dextran dose and Ussing chambers that old mice have greater intestinal permeability (specifically localized to the colon) than young mice (see figure below and Figure 3 in Thevaranjan *et al.*, 2018 10.1016/j.chom.2018.03.006):

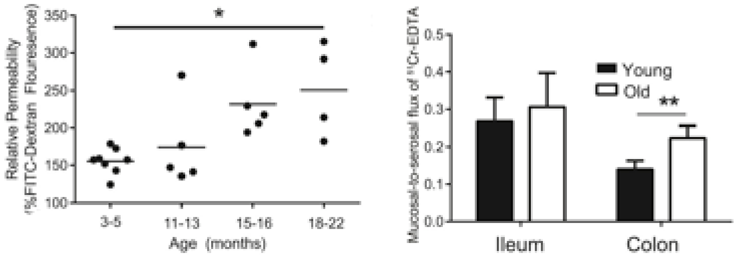

We also found using a fixed FITC-dextran dose and Ussing chambers that in non-pregnant female mice a high fat diet increases intestinal permeability within 6 weeks diet allocation, with effects also apparent after 18 weeks (see figure below and Figure 1 in Breznik *et al.*, 2023 10.1152/ajpgi.00231.2022):

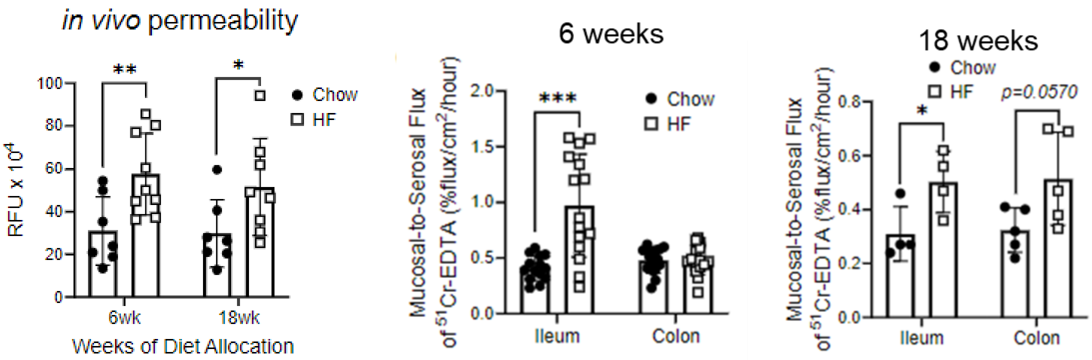

While Ussing chamber experiments were outside the scope of the current study, we are incorporating both *in vivo* and *ex vivo* assessments of permeability into further studies. For the Reviewer and Editor's interest, we have included below unpublished data from a recent study

examining changes to the gut in lactation and effects of material diet. We have performed *in vivo* permeability assessments with an adjusted dose of FITC-dextran by body weight, as in the current manuscript, with confirmation of our observations with *ex vivo* Ussing chamber assessments:

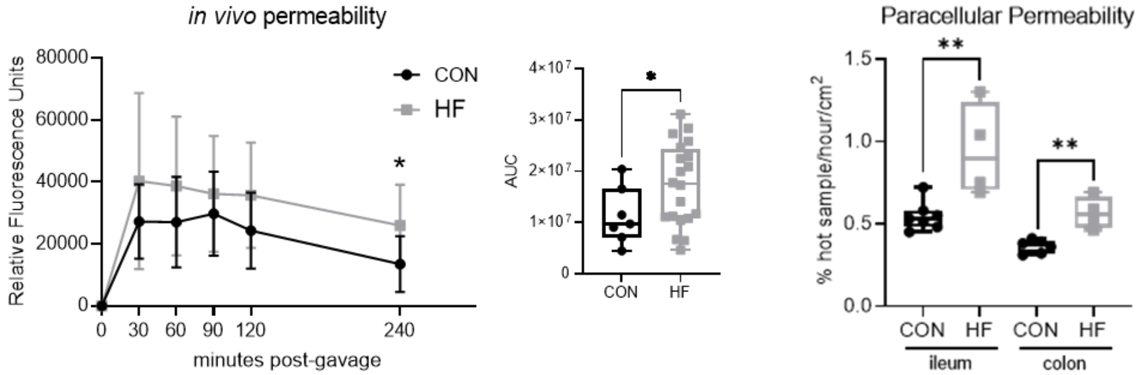

We believe that these data collectively indicate that our results in the current manuscript are not confounded by an increased dose of FITC-dextran. Furthermore, these data indicate that observations of increased permeability by *in vivo* assessments by FITC-dextran gavage align well with *ex vivo* paracellular permeability assessments using Ussing chambers, irrespective of whether a fixed or adjusted dose is used. However, from the observations of validation assessments that were performed in the current manuscript, we believe our adjusted dose approach is superior for comparisons of *in vivo* permeability when differing body weights or altered gastrointestinal motility may be present.

To acknowledge the Reviewer's suggestion, we have added text discussing limitations of our approach and recommend the use of Ussing chambers for localized assessments of intestinal permeability (lines 441-443):

> "Despite validating the use of a body-weight-adjusted method to assess *in vivo* intestinal paracellular permeability, this approach does not provide a localized assessment of intestinal permeability, as could be measured *ex vivo* using Ussing chambers (70)."

Reference 70: Thomson A, Smart K, Somerville MS, Lauder SN, Appanna G, Horwood J, et al. The Ussing chamber system for measuring intestinal permeability in health and disease. BMC gastroenterology. 2019;19(1):98.

• It would be interesting to know if the pups were weighed from each group and if differences were seen.

We describe this result on lines 193-194:

> "Average fetal weight and fetal: placental weight ratio were significantly increased in HF dams compared to CTL dams at E14.5 but not E18.5 (Table 1)."

• In the discussion, the inclusion of study limitations and relevance to human studies is not discussed.

We thank the reviewer for this suggestion, and have added the following text to the discussion (lines 440-447):

"We recognize that there are limitations to our observations that can be addressed in future studies. Despite validating the use of a body-weight-adjusted method to assess *in vivo* intestinal paracellular permeability, this approach does not provide a localized assessment of intestinal permeability, as could be measured *ex vivo* using Ussing chambers (70). We have previously assessed serum inflammatory mediators including cytokines at E14.5 and bacterial LPS and MDP at E18.5 using the same mouse model (14, 15), but we did not repeat those measures in context of our assessments of intestinal permeability and peripheral cellular immunity within this study."

References:
14. Gohir W, Kennedy KM, Wallace JG, Saoi M, Bellissimo CJ, Britz-McKibbin P, et al. High-fat diet intake modulates maternal intestinal adaptations to pregnancy and results in placental hypoxia, as well as altered fetal gut barrier proteins and immune markers. The Journal of physiology. 2019;597(12):3029-51.
15. Wallace JG, Bellissimo CJ, Yeo E, Fei Xia Y, Petrik JJ, Surette MG, et al. Obesity during pregnancy results in maternal intestinal inflammation, placental hypoxia, and alters fetal glucose metabolism at mid-gestation. Scientific reports. 2019;9(1):17621.
70. Thomson A, Smart K, Somerville MS, Lauder SN, Appanna G, Horwood J, et al. The Ussing chamber system for measuring intestinal permeability in health and disease. BMC gastroenterology. 2019;19(1):98.

Again, we would like to extend our sincere gratitude to the referees and editorial staff for their review and constructive comments. We believe that we have used their comments to make valuable improvements to the manuscript text that will benefit all readers.

Please do not hesitate to contact us if you have any further questions or concerns. We very much look forward to hearing back from PLOS ONE and hope to see our manuscript in print.

Sincerely, on behalf of the authors,

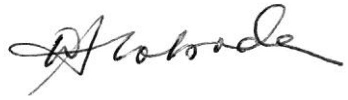

Dr Deborah M Sloboda, PhD
Professor and Associate Chair, Research
Dept of Biochemistry and Biomedical Sciences
McMaster University, Hamilton, Canada
